# Suspended Silicon Waveguide with Sub-Wavelength Grating Cladding for Optical MEMS in Mid-Infrared

**DOI:** 10.3390/mi12111311

**Published:** 2021-10-26

**Authors:** Qifeng Qiao, Haoyang Sun, Xinmiao Liu, Bowei Dong, Ji Xia, Chengkuo Lee, Guangya Zhou

**Affiliations:** 1 Department of Mechanical Engineering, National University of Singapore, Singapore 117579, Singapore; qiao@nus.edu.sg (Q.Q.); sunhaoyang@u.nus.edu (H.S.); liu.xinmiao@nus.edu.sg (X.L.); xiaji18@u.nus.edu (J.X.); 2 Department of Electrical and Computer Engineering, National University of Singapore, Singapore 117583, Singapore; eledbo@nus.edu.sg; 3 Center for Intelligent Sensors and MEMS (CISM), National University of Singapore, Singapore 117608, Singapore

**Keywords:** silicon photonics, MIR photonics, optical MEMS, photonic integrated circuit, reconfigurable photonics

## Abstract

Mid-infrared (MIR) photonics are generating considerable interest because of the potential applications in spectroscopic sensing, thermal imaging, and remote sensing. Silicon photonics is believed to be a promising solution to realize MIR photonic integrated circuits (PICs). The past decade has seen a huge growth in MIR PIC building blocks. However, there is still a need for the development of MIR reconfigurable photonics to enable powerful on-chip optical systems and new functionalities. In this paper, we present an MIR (3.7~4.1 μm wavelength range) MEMS reconfiguration approach using the suspended silicon waveguide platform on the silicon-on-insulator. With the sub-wavelength grating claddings, the photonic waveguide can be well integrated with the MEMS actuator, thus offering low-loss, energy-efficient, and effective reconfiguration. We present a simulation study on the waveguide design and depict the MEMS-integration approach. Moreover, we experimentally report the suspended waveguide with propagation loss (−2.9 dB/cm) and bending loss (−0.076 dB each). The suspended waveguide coupler is experimentally investigated. In addition, we validate the proposed optical MEMS approach using a reconfigurable ring resonator design. In conclusion, we experimentally demonstrate the proposed waveguide platform’s capability for MIR MEMS-reconfigurable photonics, which empowers the MIR on-chip optical systems for various applications.

## 1. Introduction

There has been rapid progress in the research of mid-infrared (MIR) integrated photonics in recent years [1,2]. Regarded as a technologically critical wavelength region [3], the MIR range (2~20 μm) comprises the main absorption wavelengths of many biological and chemical molecules. The so-called fingerprint region is of great importance for spectroscopic sensing. In addition, the MIR band covers several atmospheric windows (such as 3~5 μm) that are available for free-space light applications, including thermal imaging, light detection and ranging (LIDAR) systems, etc. [4,5,6,7,8,9,10,11,12,13]. Silicon photonics, a gradually matured technology in the near-infrared (NIR) telecommunication range, offers a promising solution to photonic integrated circuits (PICs) in low-cost and high-volume manufacturing [14,15,16]. 

To realize the integrated planar photonic devices in MIR, research efforts have been put into the development of waveguide platforms in different materials. The high transparency of the waveguide platform is desired for the low-loss on-chip waveguide propagation. In the past few years, high-performance MIR waveguides have been explored on silicon-on-insulator (SOI) [10,17,18,19], germanium-on-silicon (GOS) [20,21,22], sapphire-on-silicon (SOS) [23], aluminum nitride-on-insulator (ANOI) [24], and SiGe alloy-on-silicon (SGOS) [25] platforms. Silicon, as the dominating material in NIR photonics, remains attractive in the MIR. It offers low-cost manufacturing based on the mature fabrication infrastructure, as well as the bright prospect of on-chip integration of passive and active components. The inherent transparency of silicon can be up to 8.5 μm, with low absorption loss. However, the operation of the SOI platform meets the challenges in the MIR because the buried oxide (BOX) layer gradually causes a large absorption loss beyond the wavelength of 3.5 μm [2].

With the continuous development of MIR PIC components [26,27,28,29,30], reconfigurable photonic devices have gained more and more interest [31]. The capability of on-chip reconfiguration is key to enable versatile and flexible optical systems, which may not be feasible with the static PIC components. Many types of reconfiguration mechanisms have been explored in the MIR region, including mechanical motion [32,33], free carrier injection [34,35], thermo-optic tuning [36], electro-refractive switching [37], and optical phase-change media [38]. As one of the most popular approaches in NIR silicon photonics, thermo-optic tuning remains useful in the MIR. However, its operation in MIR is considered to be less efficient in terms of power consumption and response time [3]. On the other hand, another widely adopted approach in NIR, the free carrier injection, can be highly effective in a longer wavelength, but the modulation of the imaginary part of the refractive index generally induces more loss in a longer wavelength [34].

Mechanical motion enabled by optical micro-electromechanical systems (MEMS) is a mature approach for NIR PIC modulations [39,40,41,42,43,44,45]. The mechanical resonant frequency of the MEMS-enabled PIC devices typically ranges from 10 kHz to 10 MHz, which limits the on-chip actuation speed. However, effective tuning by the mechanical motion makes the optical MEMS approach effective for a wide range of waveguide material platforms and operation wavelength bands. Furthermore, MEMS tuning especially using electrostatic actuation consumes very little power compared with other approaches [39]. The electrostatic actuator relies on the electric field rather than current flow to drive the MEMS actuation. It consequently demands small power consumption. Thus, it could be an auspicious choice for the MIR reconfigurable photonics. In terms of the SOI platform, electrostatic MEMS actuators can easily be integrated into the device and substrate layer. The optical MEMS approach demands the removal of the BOX layer for movable structures; therefore, it naturally eliminates the BOX layer’s absorption loss in the MIR region, thus sustaining the competitiveness of the SOI in the MIR.

In this paper, we report the optical MEMS reconfiguration in the MIR silicon photonics (from 3.7 to 4.05 μm) on the SOI. The fully suspended silicon waveguide is achieved with sub-wavelength grating (SWG) claddings. They serve for optical mode confinement and mechanical stability of the proposed waveguide platform. In addition, SWG claddings offer a mechanically robust and optically low-loss connection between the MEMS actuator and MIR photonic waveguides. We present the simulation results on the waveguide design and illustrate the concept of optical MEMS reconfiguration. In addition, we discuss the simple fabrication process for the MEMS-integrated MIR photonic waveguide platform on the SOI, which demands a single-step dry etch only. Moreover, we demonstrate the experimental results on a few fundamental building blocks in the proposed reconfigurable waveguide platform, including the waveguide loss study, characterization of the waveguide couplers, and reconfigurable race-track ring resonators.

## 2. Design and Simulation

**Figure 1:**
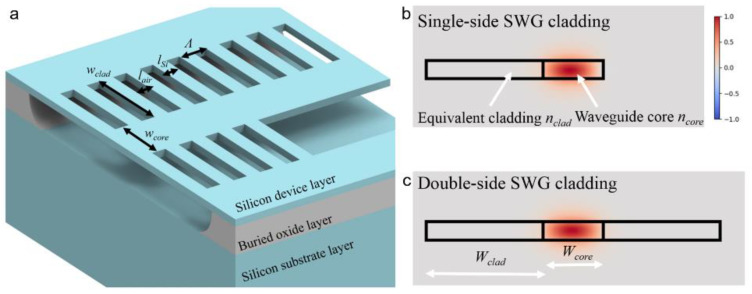
(**a**) Schematic of the suspended waveguide structure. (**b**) Mode profile (*H_y_*) of the suspended waveguide with single-side cladding. (**c**) Mode profile (*H_y_*) of the suspended waveguide with double-side cladding.

The suspended waveguide with SWG cladding designs has been adopted for silicon photonics in the MIR range (~4 μm) [46,47], and even the long-wave infrared range (~7 μm) [48,49]. In Figure 1a, we show a schematic for the geometry of the suspended waveguide used in our MEMS-integrated platform. The SOI wafer with a 500 nm thick device layer and 2 μm thick BOX layer was chosen for our MIR waveguide design (3.7~4.1 μm). In this study, we used Lumerical FDE solver for the waveguide design and simulation. Generally, the equivalent refractive index of SWG claddings is engineered to provide lateral mode confinement in the waveguide core. In addition, the lateral SWG claddings offer the mechanical robustness of the suspended waveguide, and the periodic air holes in the claddings facilitate the penetration of hydrofluoric acid (HF) during the BOX removal process.

Specifically, the waveguide structure consisted of a 1.4 μm wide waveguide core (w_core_ = 1.4 μm) and lateral SWG claddings with a width of 4 μm (w_clad_ = 4 μm). The SWG claddings comprised air holes of 0.4 μm in length (l_air_) and Si stripes of 0.2 μm in length (l_air_) in a period of 0.6 μm (Λ = l_air_ + l_air_). The proposed waveguide was designed to be single mode in TE polarization. Using such a waveguide design, the Bragg reflection can be avoided for the mode propagation and the lateral mode leakage can be reduced, thus enabling low-loss propagation. As shown in Figure 1a, the double-side SWG claddings can be converted to single-side claddings to realize the movable MEMS features. Here, we simulated the mode profile at the wavelength of 4 μm using Lumerical FDE solver. Based on the simulation results, a large overlap of 98.3% between the optical mode on the suspended waveguides with double-side cladding and single-side cladding was found (see Figure 1b,c), which guaranteed the low-loss mode conversion on the interface. 

**Figure 2:**
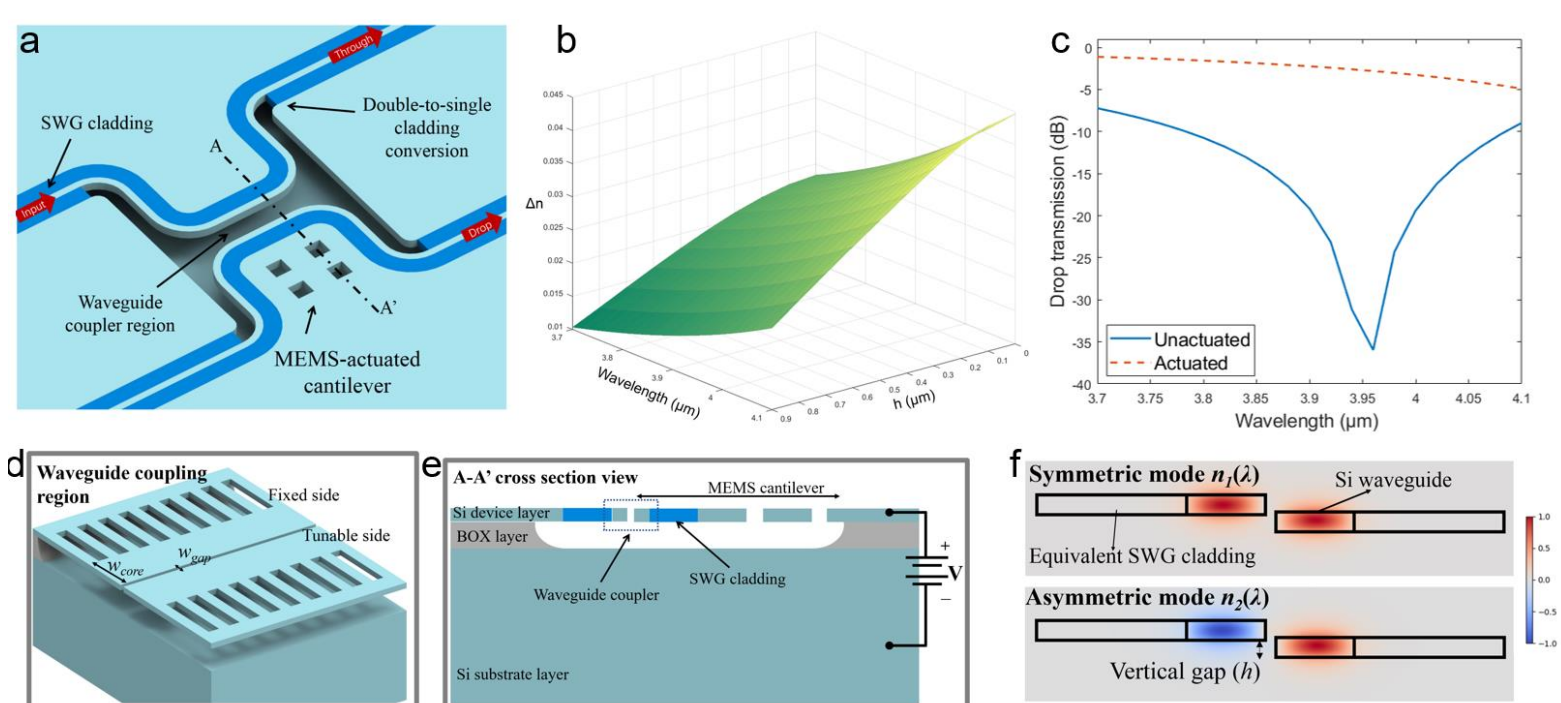
(**a**) Schematic of the reconfigurable waveguide coupler. (**b**) Simulation results of the effective indices difference Δ*n* of the symmetric and asymmetric modes. (**c**) The drop transmission spectrum of the unactuated state (0 μm gap) and actuated state (0.9 μm gap). (**d**) View of the waveguide design. (**e**) Section view to illustrate the electrostatic actuation. (**f**) Mode profile (*H_y_*) of the waveguide coupler.

Next, we take the simulation results on the tunable waveguide coupler as an example to further discuss how the proposed waveguide platform facilitates the MEMS integration. The schematic of the proposed reconfigurable waveguide coupler is shown in Figure 2a with an exaggerated coupling gap. The suspended waveguide with double-side cladding was converted to single-side cladding in the waveguide coupler region. A small coupling gap of 200 nm (w_gap_ = 200 nm) was chosen for a compact optical design. In addition, the SWG cladding and waveguide core maintained the design mentioned above. A zoomed-in view of the waveguide coupling region is shown in Figure 2d. With the HF release holes designed on the cantilever-shape Si slab, a fully suspended cantilever on the Si device layer could be achieved. Meanwhile, the cantilever shape Si slab on the other side remained rigid in a short, suspended length without the release-hole design (Figure 2e). From the cross-sectional view (Figure 2e), it can be found that the long, suspended cantilever could be electrostatically actuated downward with an applied bias voltage between the device and substrate layer, thus enabling a tunable vertical gap between the waveguide coupler. We carried out simulations on the symmetric mode and asymmetric mode of the waveguide coupler with a varying vertical gap. Similarly, the SWG claddings were modeled in an equivalent refractive index in Lumerical FDE solver (Figure 2f). The effective indices differences between the symmetric mode and asymmetric mode (Δ*n* = *n_1_* − *n_2_*) were calculated based on the simulation results. From Figure 2, it can be found that an effective tuning of Δ*n* could be achieved over a broad wavelength range (3.7~4.1 μm). From the literature, the power splitting ratio of the waveguide coupler is given by

(1) $$D=\sin^{2}{\left(\frac{\pi L\Delta n(\lambda )}{\lambda }\right)}_{}\;and\;T=\cos^{2}(\frac{\pi L\Delta n(\lambda )}{\lambda }),$$

where *D* is the drop ratio, *T* is the through ratio and *L* is the coupling length. The maximum vertical gap of the waveguide coupler can be estimated to be 0.9 μm based on the MEMS cantilever electrostatic pull-in model and the 2 μm BOX thickness [50]. *L_π_(λ)*, which is defined as the coupling length required for a complete power transfer from one waveguide to the other, is critical for the waveguide coupler performance. Based on our simulation results and Equation (1), the *L_π_* at the wavelength of 3.95 μm was found to be 57 μm. The simulated drop transmission spectrum is shown in Figure 1c. With an MEMS-actuated vertical gap of 0.9 μm (Figure 1c), the effective tuning of Δ*n* could reach 0.0224, which potentially achieved a reconfigurable optical power splitter and switch on the proposed waveguide platform. 

**Figure 3:**
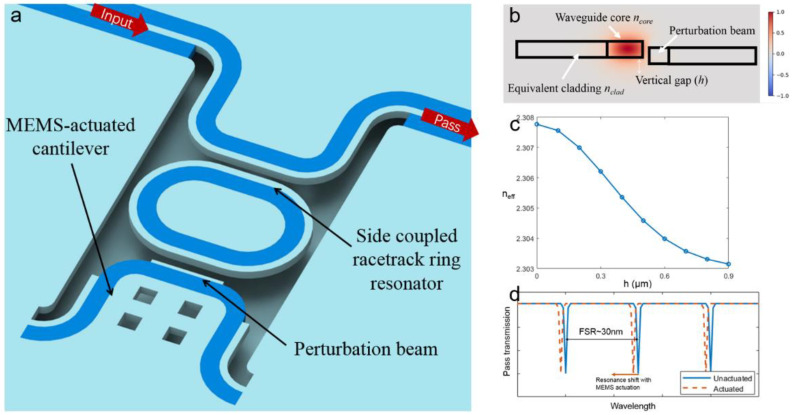
(**a**) Schematic of the reconfigurable ring resonator. (**b**) Mode profile (*H_y_*) of the perturbed waveguide mode. (**c**) Simulation results of the effective index n_eff_ under perturbation at the wavelength of 3.9 μm. (**d**) Schematic pass transmission spectrum of the ring resonator under the MEMS tuning.

Furthermore, we propose a reconfigurable ring resonator concept, which shows the versatile design potential of the proposed reconfigurable waveguide platform. As shown in Figure 3a, the reconfigurable device consisted of a side-coupled racetrack ring resonator and a tunable perturbation beam with an MEMS actuator. The double-side SWG cladding was converted to a single-side to allow coupling between the bus waveguide and ring resonator. The width of the bus waveguide in the coupling region was tapered to the same as the waveguide width of the ring resonator, which was 1.3 μm. The tapered design can enhance the coupling condition. The ring resonator was connected to a fixed Si slab via the SWG claddings, which followed the same SWG design as mentioned above. On the other side, a perturbation beam with a width of 0.6 μm was placed close to the ring resonator. The width of the perturbation beam was designed such that it did not support any propagation mode. As a result, the perturbation beam only changed the field profile and effective index of the waveguide mode in the ring resonator at the affected region. With the vertical actuation of the perturbation beam, it induced a phase shift or an effective length change to the ring resonator, thus tuning the spectral characteristics of the resonance. To validate the proposed reconfiguration mechanism, we firstly designed a ring resonator with 30 nm free spectra range (FSR). From the literature [51], the FSR of a ring resonator can be given by

(2) $$FSR=\frac{\lambda^{2}}{n_{g}L}$$

where *n_g_* is the group index and *L* is the round-trip length. From the FDE simulations, *n_g_* at the wavelength of 3.8 μm was around 3.92. Thus, the round-trip length was designed to be 123 μm to satisfy the desired FSR. The racetrack ring resonator design was adopted to reduce the fabrication errors’ effect on the ring performance. To avoid bending loss on the ring resonator, a bending radius of 10 μm was given, and the remaining part was straight to meet the 123 μm round trip length. Details of the design parameters can be found in the experimental section. To validate its perturbation on the field profile of the ring resonator waveguide, we carried out simulations with the geometry of the ring resonator waveguide and perturbation beam (as shown in Figure 3b). The effective index of the perturbed mode was calculated. By moving the perturbation beam slightly downwards using an MEMS actuator, the effective index decreased from 2.3078 to 2.3031. From the literature [51], the resonance wavelength of a ring resonator can be given by

(3) $$\lambda_{res}=\frac{n_{eff}L}{m},m=1,2,3...$$

From Equation (3), it can be found that the MEMS actuation of the perturbation beam will lead the resonance to a shorter wavelength (Figure 2d).

In this section, we illustrate the implementation of optical MEMS reconfiguration on the suspended waveguide platform using simulation results. A few merits of the proposed reconfiguration approach using the SWG design and MEMS actuation can be found. Firstly, the insertion loss of the MEMS actuator could be minimized because it was connected to the photonic waveguides via the SWG claddings. At the same time, the dense SWG structures also offered great mechanical robustness. Secondly, the mechanical motion enabled effective tuning of the waveguide component in the MIR range, which was investigated by simulations. Thirdly, with the dedicated simultaneous engineering on the optical and mechanical design, the proposed approach could enable versatile reconfigurable designs.

## 3. Fabrication and Testing Setup

**Figure 4:**
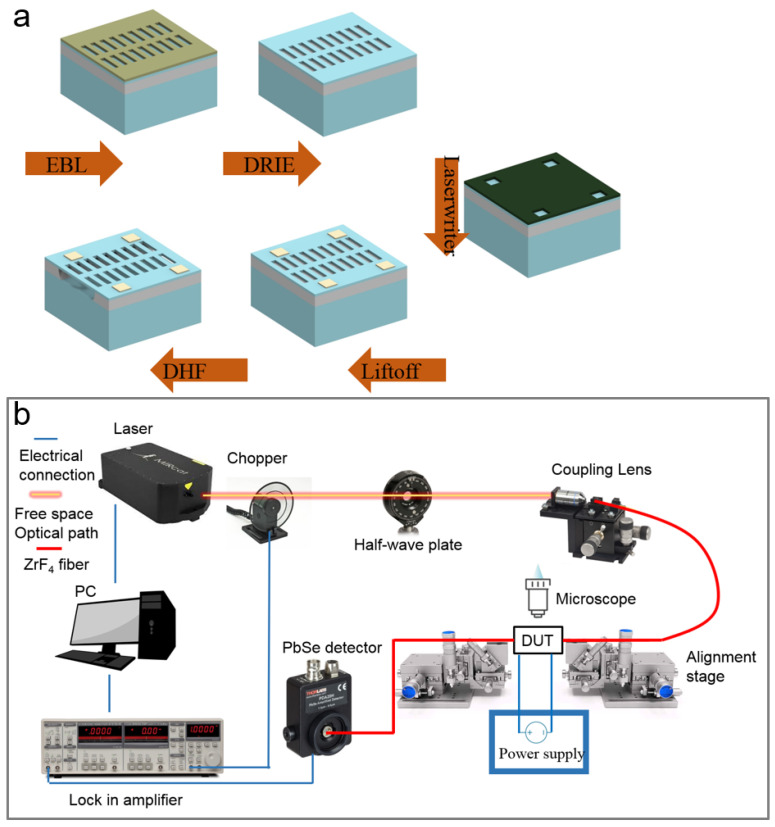
(**a**) Fabrication process flow. (**b**) Experimental setup.

The experimental devices were fabricated in a compatible process with silicon photonics foundry. In the first step, we diced the 8-inch SOI wafer (500 nm thick Si and 2 μm thick BOX) into 1.5 cm × 1.5 cm dies. Then, we worked on the nanofabrication process on a die scale (Figure 4a). We firstly used standard electron beam lithography (EBL) to pattern the dielectric features (including the photonic design and MEMS structure) on the e-beam resist (ZEP 520A). Next, deep reactive ion etching (DRIE) was adopted to thoroughly etch the silicon, thus transferring the EBL pattern to the Si layer. After the removal of the e-beam resist, we used standard optical lithography (Laserwriter) to define the electrode pattern on the photoresist (AZ1512). The metal of 5 nm thick Cr and 50 nm thick Au was deposited using a thermal evaporator, which was followed by a lift-off process (rinsing in 65 °C acetone). Finally, the die was put into the diluted HF (DHF) to remove the BOX layer. A ratio of 1:4 between the 49% HF and DI water was used here. After 1.5 h of DHF release, the die was transferred to the critical point dryer to remove the liquid and avoid stiction. The proposed reconfigurable waveguide platform requires a simple fabrication process. The MEMS and photonic patterns on the Si layer could be achieved together with single-step lithography and following dry etch. 

The experimental testing setup is depicted in Figure 4b. A quantum cascaded laser (Daylight, MIRcat-1200, San Diego, CA, USA) was used as the MIR light source. A mechanical disk chopper (Stanford Research SR540) was placed in the free-space light path and connected to the lock-in amplifier (SR830) as the reference frequency. After the chopper, a half-wave plate (Thorlabs WPLQ05M-4000, Newton, NJ, USA) was used to adjust the polarization. Through the ZnSe lens, the free-space laser beam was focused on a ZrF_4_ fiber. We used the fiber-to-chip grating couplers to input the light and collect the output. The fiber ends were mounted on a pair of mechanical positioners at a 10° tilt. Using the vertical microscope, we achieved alignment between fiber ends and grating couplers. The output light was sent to a photodetector (Thorlabs PDA20H) that was connected to the lock-in amplifier as an input. The bias voltage was applied to the on-chip device via the microprobe positioners. The lock-in amplifier was used to reduce environmental noise in the testing system.

## 4. Experimental Results

### 4.1. Waveguide Loss Study

**Figure 5:**
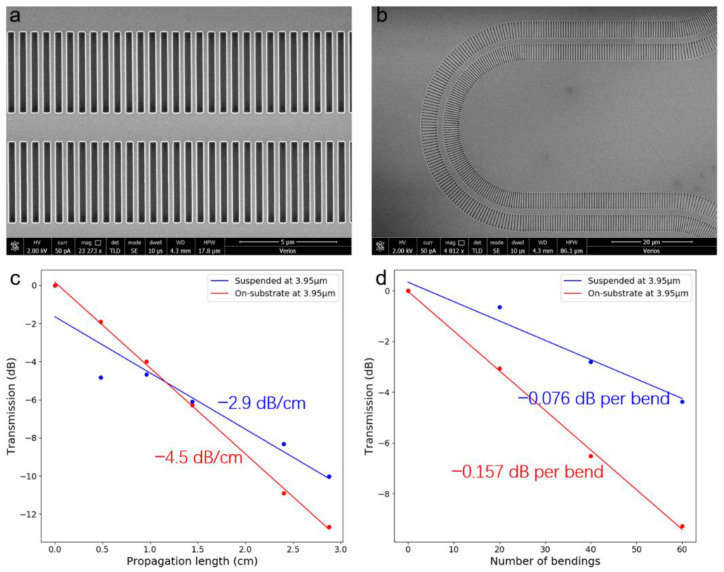
(**a**,**b**) SEM photos of the straight and bending waveguide. (**c**) Measurements of the propagation loss. (**d**) Measurements of the bending loss.

We experimentally investigated the propagation loss and bending loss of the suspended waveguide with SWG claddings. A sub-wavelength grating coupler design operating around a wavelength of 3.95 μm is adopted for the light in and out of the chip [52]. The waveguide devices were measured before and after DHF removal, which are denoted as on-substrate and suspended devices, respectively. The cut-back method was used to determine the waveguide loss. For the propagation loss, the transmission measurements on several propagation lengths from 0.6 cm and 3.48 cm at the wavelength of 3.95 μm were used for analysis. Similarly, the bending loss was determined with the transmission measurements on a varying number of bendings (20 μm radius) from 20 to 80 at a wavelength of 3.95 μm. As result, the propagation loss could be reduced from −4.5 dB/cm to −2.9 dB/cm with the removal of the BOX layer (Figure 5c), and the bending loss could be reduced from −0.157 dB each to −0.076 dB each (Figure 5d). It can be found that the waveguide loss could be greatly reduced after the BOX removal. This can be attributed to the elimination of oxide absorption loss. In addition, the suspended waveguide structure also offers more symmetric mode confinement compared with the on-substrate one. The reported waveguide loss is comparable to the study in [47]. 

### 4.2. Waveguide Coupler Characterization

**Figure 6:**
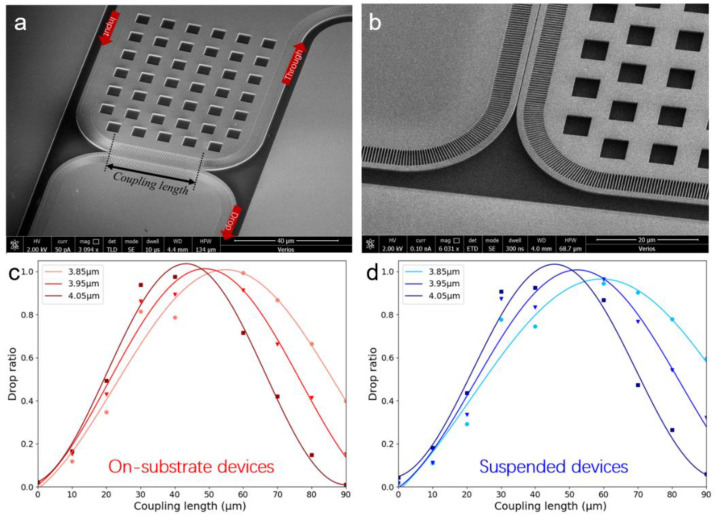
(**a**) SEM photo of an on-substrate waveguide coupler. (**b**) SEM photo of a suspended waveguide coupler. (**c**) Measured drop ratio of the on-substrate devices. (**d**) Measured drop ratio of the suspended devices.

We experimentally investigated the waveguide coupler. A bending radius of 20 μm was used in our waveguide design. The geometric parameters of the waveguide coupler are discussed in Section 2. Waveguide coupling length ranging between 20 and 90 μm was fabricated and tested before and after BOX removal. SEM photos of the directional couplers are shown in Figure 6a,b. The suspended feature of the waveguide coupler is clearly shown in Figure 6b. The directional couplers were designed as 2 × 2 ports. For each device testing, one input (*I*) port and a corresponding through (*T*) port were first best aligned. Then, the optical transmission of the through port was tested. Subsequently, the input fiber end was fixed, and the output fiber end was moved to the drop (*D*) port for testing. It was assumed that *I = D + T* and the drop ratio was defined as *D*/*I*. In this way, the power splitting ratio could be determined. The relationship between drop ratio and coupling length is shown in Figure 6c,d. The sine square function fitted the testing results well, which proves the optical performance of the devices. In addition, it can be found that the BOX removal leads the *L_π_(λ)* to a slightly longer wavelength. For the wavelength of 3.95 μm, *L_π_* of the suspended waveguide coupler was around 50 μm, excluding the curved part, which is slightly shorter than the simulated results (57 μm). This can be attributed to the extra coupling caused by the curved parts and fabrication imperfections. In addition, the insertion loss of the waveguide couplers was characterized. The total transmission *(D + T)* values of nine suspended waveguide couplers were compared with the optical transmission from a reference straight waveguide, which is similar to the total propagation length of the waveguide coupler device. An insertion loss of −1.66 ± 0.69 dB was found at a wavelength of 3.95 μm. The insertion loss can be attributed to the fabrication imperfection and the mode mismatch at the cladding conversion interface. 

### 4.3. Reconfigurable Race-Track Ring Resonator

**Figure 7:**
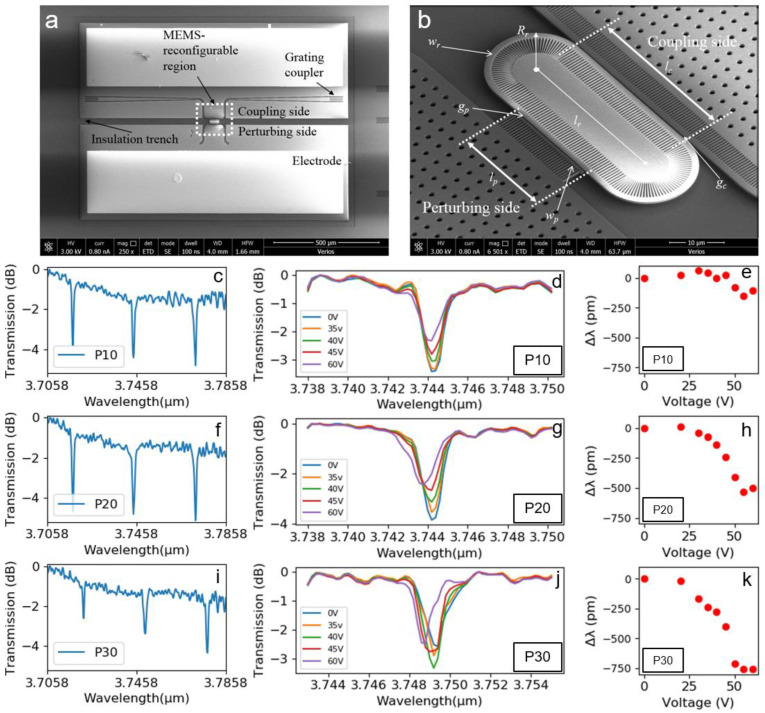
(**a**,**b**) SEM photos of the experimental devices. (**c**–**e**) Measured results of P10. (**f**–**h**) Measured results of P20. (**i**–**k**) Measured results of P30.

We experimentally implemented the concept of the reconfigurable ring resonator discussed in Section 2. As shown in Figure 7a, the experimental device consisted of the coupling side, the perturbing side, and the race-track ring resonator in the middle. At the coupling side, the bus waveguide was attached to the cantilever-shaped Si slab with single-side SWG claddings to enable light coupling with the ring resonator. At the perturbing side, a narrow Si beam was attached to the movable MEMS cantilever to perturb the evanescent field of the ring resonator waveguide. With the insulation trenches, the movable cantilevers on the coupling and perturbing sides could be electrically separated and had individual electrical potentials. In this study, we only investigated the mode perturbation. Thus, the electrode on the coupling side and the Si substrate were grounded. Meanwhile, the bias voltage was applied on the perturbing side to offer the electrostatic force for vertically downward actuation. Design parameters are denoted in Figure 7b. Specifically, w_r_ = 1.3 μm, R_r_ = 10 μm, l_r_ = l_c_ = 30 μm, the gap on the coupling side g_c_ = 400 nm, the gap on the perturbing side g_p_= 260 nm, the perturbation beam width w_p_ = 600 nm. The round-trip length of the racetrack ring resonator was around 123 μm to meet our design target. The bus waveguide on the coupling side was tapered from 1.4 μm to 1.3 μm to enhance the coupling. The width of the perturbation beam was designed to be the mode cut-off condition. Thus, there mode coupling was not induced on the perturbing side. The suspended MEMS cantilever length on the perturbing side was designed to be 30 μm, and the pull-in voltage could be estimated to be 75 V [50]. Three devices with varying perturbation beam lengths, 10 μm, 20 μm and 30 μm, were investigated and subsequently denoted as P10, P20 and P30, respectively. 

We firstly investigated the spectral characteristics of these three ring resonators without MEMS actuation. The swept spectra in a 0.2 nm resolution of these three ring resonators are shown in Figure 7c,f,i, respectively. The Lorentz fitting was used to fit the resonance dips and extract the spectral characteristics. The Q factor, extinction ratio (ER), and FSR of these devices are summarized in Table 1. Next, we implemented MEMS actuation on each reconfigurable ring resonator using a bias voltage. The swept spectra were obtained under each static bias voltage from 0 V to 20 V, 30 V, 35 V, 40 V, 45 V, 50 V, 55 V and 60 V. A single resonance dip within the FSR from each device was selected to monitor the spectral response to the applied bias voltage. Testing results are presented in Figure 7d,g,j for the three devices. It could be found that the Q factor of the resonance was barely tuned for these three devices. To quantitively evaluate the reconfiguration capability of the proposed scheme, Lorentz fitting was used on the measured resonance dips under the bias voltage actuation. The resonance wavelength shifts (Δλ) concerning the applied voltage are shown in Figure 7e,h,k. It can be found that the device P30 with a perturbation beam length of 30 μm had the largest reconfiguration capability among the three designs. With an applied bias voltage from 0 V to 60 V, a resonance wavelength shift of 800 pm could be achieved. In comparison, the P20 and P10 devices offered a maximum resonance wavelength shift of 500 pm and 100 pm, respectively. Compared with the simulation results, the experimental FSR 27.4 nm slightly deviated from 30 nm, which can be mainly attributed to fabrication error. The blue shift of the resonance caused by MEMS actuation in the experiments was in accordance with the simulation results. 

## 5. Discussion

As a result, we present the loss study on the suspended waveguide with SWG claddings operating at the MIR wavelength range (3.7~4.1 μm). A propagation loss of −2.9 dB/cm and bending loss of −0.076 dB are each reported. With the optimization of the nanofabrication process [46], the sidewall roughness of the waveguide can be further improved, thus reducing the waveguide loss. The low-loss feature of the proposed waveguide platform on the SOI could be an auspicious choice for the realization of MIR PICs.

In addition, we experimentally characterized the waveguide couplers based on the proposed waveguide platform. An *L_π_* of 50 μm at the wavelength of 3.95 μm was obtained, and insertion loss of the movable device was 1.66 ± 0.69 dB. The measured *L_π_* promises a compact device footprint of the reconfigurable waveguide coupler. The low insertion loss shows the easy integration of the MEMS actuator into the suspended waveguide platform using the SWG design. With advanced nanofabrication technology, the coupling gap and waveguide surface roughness could be further reduced, thus enabling a more compact footprint and lower insertion loss [53,54]. 

Moreover, we experimentally validated the reconfigurable race-track ring resonator based on the proposed approach. The electrostatic MEMS cantilever actuator was used on the SOI to realize mode perturbation of the ring resonator. In the experiment, we obtained a maximum resonance wavelength shift of 800 pm with 60 V bias applied. To further enhance the reconfiguration capability, engineering efforts should be put on both the photonic and MEMS design. Dedicated optimization could be carried out to enhance the optical performance of the ring resonator. Other MEMS actuation architectures can be considered to enable a large displacement of photonic components, such as the in-plane comb drive and 3D integration [33,55]. 

Overall, we have proposed and validated a promising solution to MIR reconfigurable photonics. Combining the MEMS and photonic waveguide via the SWG design on the SOI wafer, the proposed solution features low costs, easy integration, efficient energy consumption and effective tuning. The proposed platform offers great potential to realize versatile and powerful MIR on-chip systems, thus facilitating the implementation of spectroscopy and remote sensing on MIR PIC chips.

## Figures and Tables

**Table 1:** Spectral characteristics of the static ring resonators.

Device	Q	ER (dB)	FSR (nm)
P10	3670	2.75	27.2
P20	2900	3.33	27
P30	3290	1.96	27.4

## Data Availability

Data are available from the corresponding authors C.L. and G.Z. upon reasonable request.
